# Land Use and Land Cover Classification Meets Deep Learning: A Review

**DOI:** 10.3390/s23218966

**Published:** 2023-11-03

**Authors:** Shengyu Zhao, Kaiwen Tu, Shutong Ye, Hao Tang, Yaocong Hu, Chao Xie

**Affiliations:** 1 College of Mechanical and Electronic Engineering, Nanjing Forestry University, Nanjing 210037, China; 2 School of Electrical Engineering, Anhui Polytechnic University, Wuhu 241000, China; 3 College of Landscape Architecture, Nanjing Forestry University, Nanjing 210037, China

**Keywords:** deep learning, LULC, image classification, remote sensing

## Abstract

As one of the important components of Earth observation technology, land use and land cover (LULC) image classification plays an essential role. It uses remote sensing techniques to classify specific categories of ground cover as a means of analyzing and understanding the natural attributes of the Earth’s surface and the state of land use. It provides important information for applications in environmental protection, urban planning, and land resource management. However, remote sensing images are usually high-dimensional data and have limited available labeled samples, so performing the LULC classification task faces great challenges. In recent years, due to the emergence of deep learning technology, remote sensing data processing methods based on deep learning have achieved remarkable results, bringing new possibilities for the research and development of LULC classification. In this paper, we present a systematic review of deep-learning-based LULC classification, mainly covering the following five aspects: (1) introduction of the main components of five typical deep learning networks, how they work, and their unique benefits; (2) summary of two baseline datasets for LULC classification (pixel-level, patch-level) and performance metrics for evaluating different models (OA, AA, F1, and MIOU); (3) review of deep learning strategies in LULC classification studies, including convolutional neural networks (CNNs), autoencoders (AEs), generative adversarial networks (GANs), and recurrent neural networks (RNNs); (4) challenges faced by LULC classification and processing schemes under limited training samples; (5) outlooks on the future development of deep-learning-based LULC classification.

## 1. Introduction

Land use and land cover (LULC) is the expression of the transformation of the natural ecosystem of land into an artificial ecosystem and the naturally occurring or human-induced cover on the surface. It serves an important role in the fields of disaster management, urban planning, environmental protection, and agricultural production, and has been a popular theme in Earth observation research. Remote sensing images have become the main data source for LULC classification due to their advantages, such as wide coverage and continuous monitoring to obtain time series data. Since the introduction of deep learning, excellent results have been achieved in the field of image processing, and thus, LULC classification has become a popular research topic [1,2,3].

The initial classification technique was visual interpretation classification, which was judged by the expertise of the interpreter. The advantage is that the accuracy is high, generally higher than the computer classification accuracy, so the visual interpretation classification is still applied to high-precision, high-resolution remote sensing image classification [4]. However, the biggest disadvantage is poor repeatability and poor timeliness. With the development of computer technology, machine learning is widely used in LULC classification. Traditional classification techniques are divided into two types: unsupervised and supervised classification. Unsupervised classification is represented by K-Means and Expectation Maximization, which can distinguish the categories for data processing; however, the attributes of the classification results are uncertain. Common supervised classification algorithms include support vector machine (SVM), decision tree, maximum likelihood classification, and so on [5,6,7,8]. The parameters of the discriminant function are derived from the known image element data, and then the discriminant function is used to classify the unknown image elements. Traditional classification techniques are characterized by good repeatability and timeliness compared to visual interpretation; however, the accuracy of the classification will be greatly reduced after changing the data or study area [9].

In contrast to classic machine learning algorithms, deep learning (DL) demonstrates unique advantages in image classification. While traditional machine learning algorithms require the manual design of features for classification tasks, deep learning eliminates the need for manual intervention; it automatically learns and extracts features relevant to the target task, and this automatic feature extraction capability endows deep learning models with strong robustness and makes it easier to migrate the models across different datasets [10,11,12,13,14]. Deep learning algorithms can learn from large-scale data and discover potential patterns and regularities, thus improving the accuracy and effectiveness of LULC classification [9].

**Figure 1:**
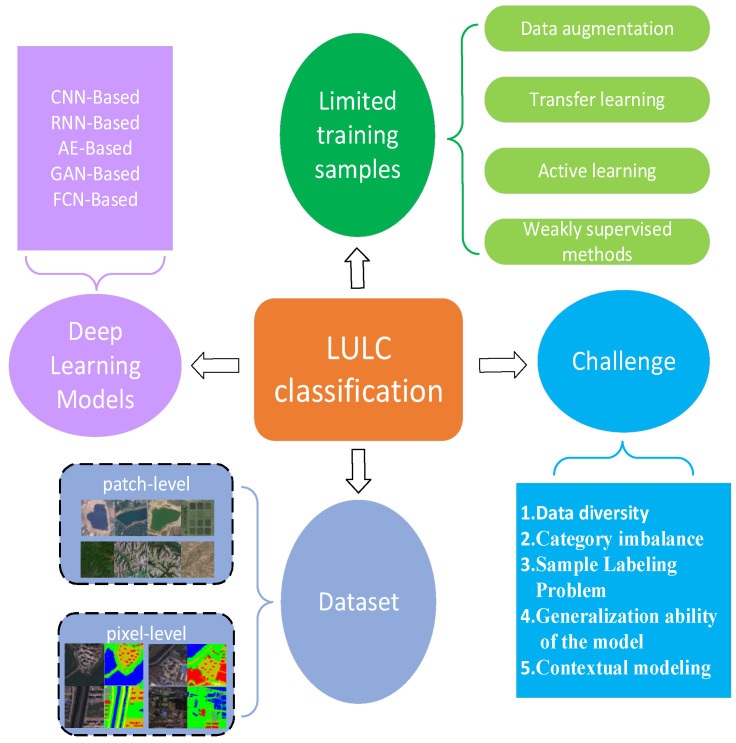
LULC classification general framework diagram.

The rest of this paper is organized as follows: Section 2 describes the composition and construction of five commonly used deep learning models (CNN, RNN, AE, GAN, and FCN). Section 3 systematically summarizes the two benchmark datasets (patch-level and pixel-level) used for LULC classification, along with performance metrics. Section 4 describes the LULC classification based on a deep learning approach. Section 5 lists the challenges of LULC classification, such as category imbalance, sample labeling problems, etc. Section 6 discusses four solutions to the LULC classification problem with limited training samples (transfer learning, data augmentation, active learning, weak supervision methods). Section 7 provides an outlook for future research on deep learning in LULC classification. It concludes in Section 8. The general framework of this review is shown in Figure 1.

## 2. Typical DL Models

Deep learning [15], a unique branch in the machine learning field, solves complex problems by performing computations through multiple layers of successive neural networks, i.e., mimicking human brain mechanisms to interpret data. The key to DL is to build deep neural networks by increasing the number of network layers so as to obtain a powerful performance that allows the algorithm to cope with more complex tasks. Moreover, DL plays a crucial role in processing and analyzing large-scale data by utilizing efficient algorithms for unsupervised or semi-supervised feature learning, as well as hierarchical feature extraction, thus getting rid of the tedious process of manually extracting features [16]. Commonly used DL models are convolutional neural networks, generative adversarial networks, recurrent neural networks, autoencoders, and full convolutional neural networks.

### 2.1. Convolutional Neural Networks

**Figure 2:**
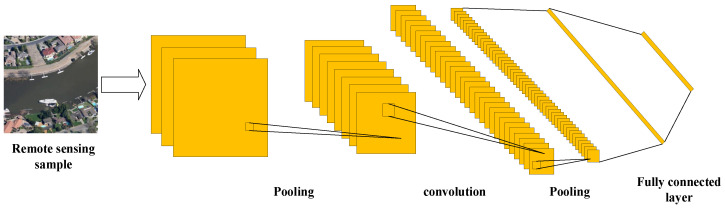
Graphical representation of CNN.

Convolutional neural network (CNN) is a neural network with convolutional operational feedback and deep structure in the network. Simply put, it is a system that simulates the vision of the human brain. It is a multilayer neural network structure consisting of three parts: input layer, intermediate layer, and output layer, where the intermediate layer contains some hidden layers [17,18]. After convolutional operations, the multilayer neural network combines the main features of the image to gradually generate high-level features. This process extracts features with high-level semantic information from local information, realizing multi-level information transfer and gradual integration. By combining feature extraction with classification recognition, the image recognition task can be carried out. The intermediate layer of CNN is also composed of three parts: convolutional layer, pooling layer, and fully connected layer. It is developed and researched based on neural networks. Compared to traditional neural networks, the unique feature of CNN is the convolution operation in the convolution layer. The input image goes through the convolutional layer to extract local features, and then goes to the pooling layer to downsize the extracted local features where the two layers, the convolutional layer and pooling layer, can be stacked multiple times repeatedly. The input 2D image information is transferred layer by layer to the fully connected layer to associate the high-level features obtained from the learning of convolutional and pooling layers, with the output categories making the final classification decision and finally outputting the result [19,20,21,22]. Figure 2 shows the graphical representation of CNN.

### 2.2. Recurrent Neural Networks

**Figure 3:**
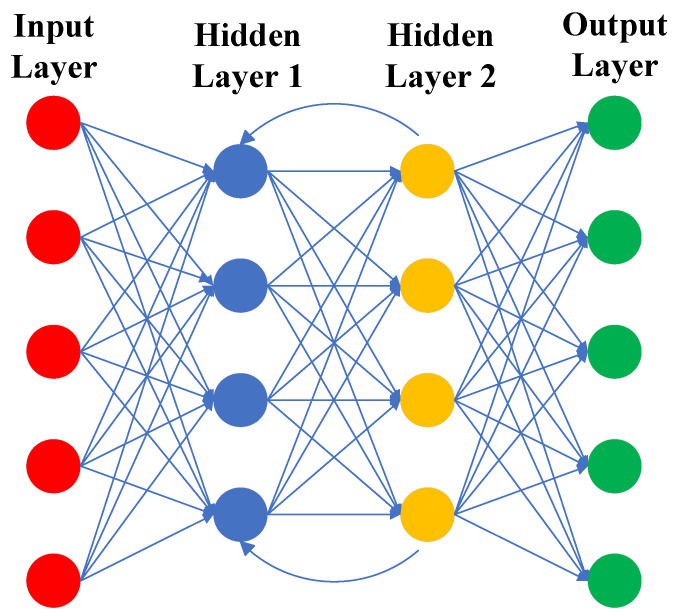
Graphical depiction of RNN.

Recurrent neural network (RNN) [23,24,25], as one of the branches of deep learning networks, usually refers to temporal recurrent neural networks, which are mainly used to process sequential data, with the aim of associating the current output of a sequence with the previous information to form a recurrent connection. The most important feature of RNN is the hidden state throughout the input and output of the network. It is used as an input to the RNN along with the input vectors, which are updated and then used as an output to the RNN along with the output vectors [26,27]. At this point, the updated hidden state that is output will be used as part of the next input, thus preserving the previous information. Remote sensing images usually contain both temporal and spatial information, and RNNs are often applied to LULC classification due to their powerful temporal processing capabilities. Figure 3 shows the graphical depiction of RNN.

### 2.3. Generating Adversarial Networks

**Figure 4:**
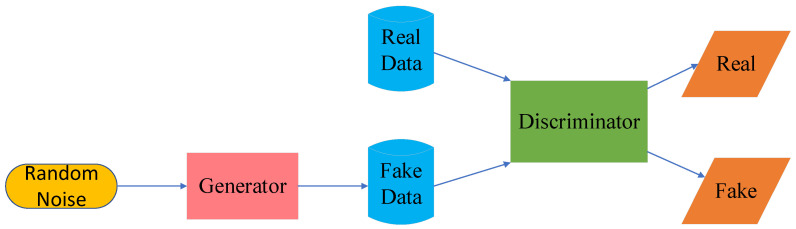
Graphical representation of GAN.

Generative adversarial network (GAN) is one of the most promising unsupervised learnings in recent years. It contains two kinds of neural networks, generator network and discriminator network, and the two neural networks learn against each other. During the training process, the generator constantly adjusts its parameters to allow the generated samples to fool the discriminator [28]. The discriminator, to more accurately determine whether the samples are real samples or generator samples, also constantly adjusts the parameters. In this way, in iterative optimization, the final generator-generated samples are realistic enough so the discriminator cannot accurately distinguish. GAN, due to its unique adversarial learning mechanism, can solve the problems caused by category imbalance, cross-region adaptive learning, and data augmentation, thus improving the accuracy and robustness of the network. Figure 4 shows the graphical representation of GAN [29].

### 2.4. Autoencoder

Autoencoder (AE) is an unsupervised deep learning model consisting of an encoder and a decoder. The functionality is similar to PCA; however, in contrast to PCA, AE not only handles linear mapping but also performs nonlinear mappings for better performance. The input data will be mapped to a lower dimension than the original data for the coded representation while the encoder reconstructs the data as much as possible so that the coded representation maps back to the original dimension. Thus, the ultimate goal of AE is to reconstruct the output similar to the original data, i.e., to minimize the reconstruction error of the decoder as much as possible [30]. When the number of layers of AE is increased to multiple layers, it is a stacked autoencoder (SAE), also called a depth autoencoder. It consists of an input layer, a hidden layer stack, and an output layer. The training process is divided into two phases, as in AE: the encoding phase and decoding phase. It can be expressed by the following equation:

(1) $$\left\{\begin{array}{c} Hidden=AF\left(W_{\mathrm{Hidden}\;}\times {x+B}_{\mathrm{Hidden}\;}\right)\\ Output=AF\left(W_{\mathrm{Output}\;}{\times x+B}_{\mathrm{Output}\;}\right) \end{array}\right.$$

**Figure 5:**
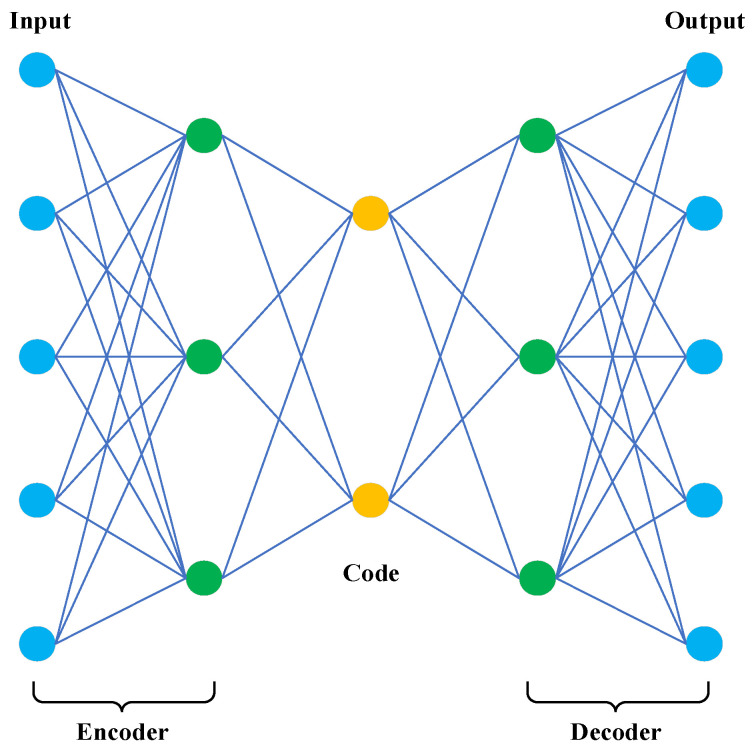
Graphical depiction of AE.

W_Hidden_ and W_output_ denote the weight of input to hidden and hidden to output, respectively. B_hidden_ denotes hidden layer bias and B_output_ denotes output layer bias. AF is the activation function. SAE is widely used in LULC classification due to the fact that it can help to extract the low-dimensional features in remote sensing images and capture the key information. Figure 5 shows the graphical depiction of AE.

### 2.5. Fully Convolutional Neural Networks

**Figure 6:**
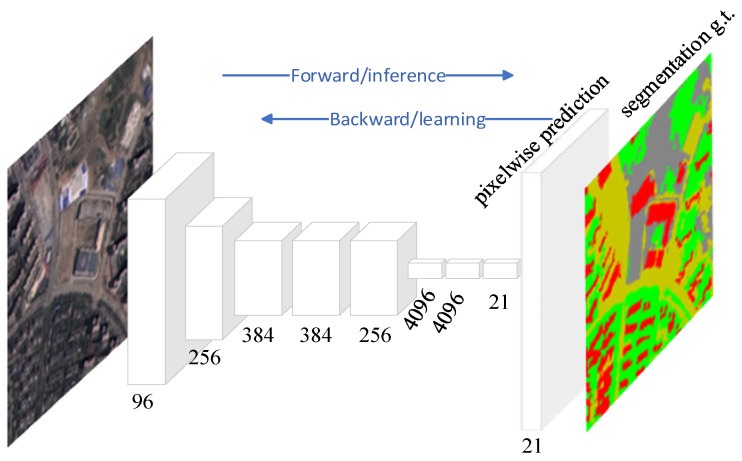
Graphical representation of FCN.

Fully convolutional neural networks (FCNs) belong to a special variant of CNNs and are mainly used for image semantic segmentation. Unlike CNN, it does not contain fully connected layers, but replaces fully connected layers with fully convolutional layers. Some FCNs also contain some local perceptual layers, e.g., transposed convolutional layers, upsampling layers, etc. Unlike traditional CNN-based semantic segmentation methods that take image chunks as input, FCN samples a more direct approach, instead taking the entire image as input and manually labeling the graph as output. This end-to-end training approach greatly improves the computational efficiency of the network as well as more accurate segmentation results, making FCN stand out in semantic segmentation tasks. FCN can be a favorable tool for LULC classification because it not only provides pixel-level prediction, it also captures the spatial information in remote sensing images well in order to achieve multi-scale feature learning and other advantages, so as to achieve efficient and accurate classification [31,32]. Figure 6 shows an illustration of FCN, where an input image of arbitrary size is first subjected to feature extraction through multiple convolutional and pooling layers in which the feature map resolution is gradually reduced while higher semantic features are obtained. Secondly, pixel level segmentation prediction is generated by transposed convolutional layer operations to restore the image resolution to the input state. Finally, the image is outputted.

## 3. Datasets and Performance Metrics

In deep learning, the accuracy of classification relies heavily on high-quality datasets as well as proper class categorization. Especially with the introduction of CNNs, it becomes crucial to have a large amount of training data to support the training of the network. With the continuous development of deep learning, various deep neural network models are applied to LULC classification, which makes comparing the gaps between models a research hotspot, so it is important to construct an open-source sample dataset. In recent years, researchers and organizations have released a variety of LULC classification datasets. These datasets cover a rich variety of data samples, including multi-scale images, data collected by different sensors, and multimodal features, providing a more reliable basis for data comparison in related studies. These datasets are broadly categorized into two types: patch-level datasets and pixel-level datasets. Patch-level datasets refer to the assignment of a fixed-size image to a specific feature class, and are mostly used for remote sensing image scene classification. Pixel-level samples, on the other hand, where each pixel point is considered a sample and assigned to the respective category, are mostly used for semantic segmentation of remote sensing images.

### 3.1. Pixel-Level Datasets

Pixel-level datasets are used for LULC classification whose ultimate goal is the segmented map, i.e., land cover mapping. Such pixel-level datasets are mostly used as training semantic segmentation models, which is a huge amount of work involved in segmenting an image into several constituent regions with specific labeling of all pixels covered by the segmented regions. Therefore, producing such datasets can take a lot of time and labor on the part of the researcher. Four pixel-level benchmark datasets for LULC classification are described below.

**Figure 7:**
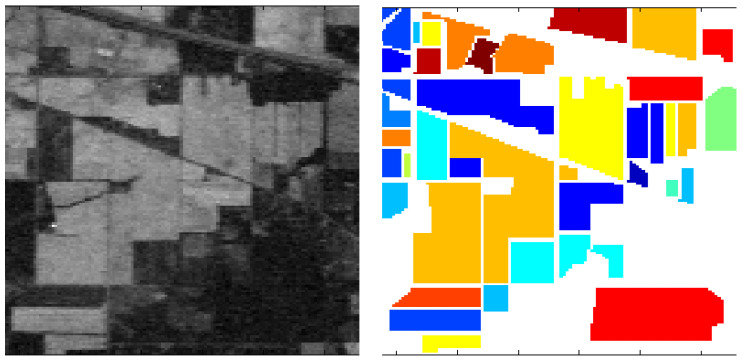
Schematic diagram of the Indian Pines.

**Figure 8:**
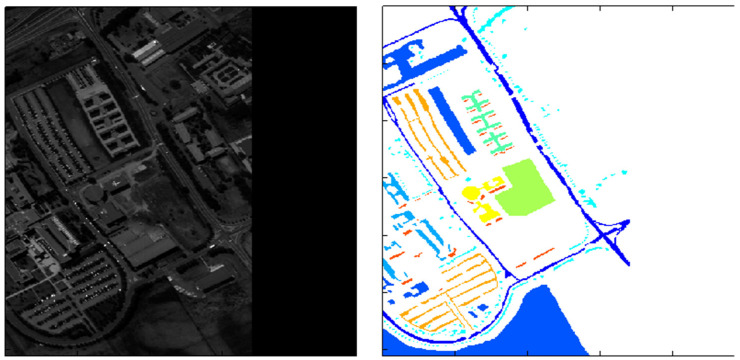
Schematic diagram of Pavia University.

**Figure 9:**
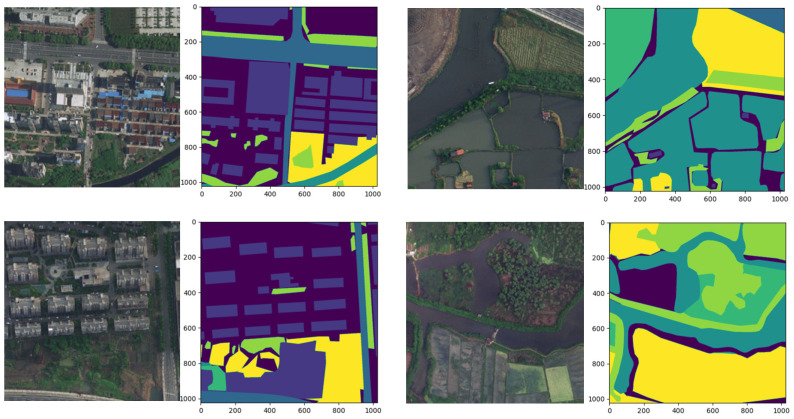
Schematic diagram of LoveDA.

**Figure 10:**
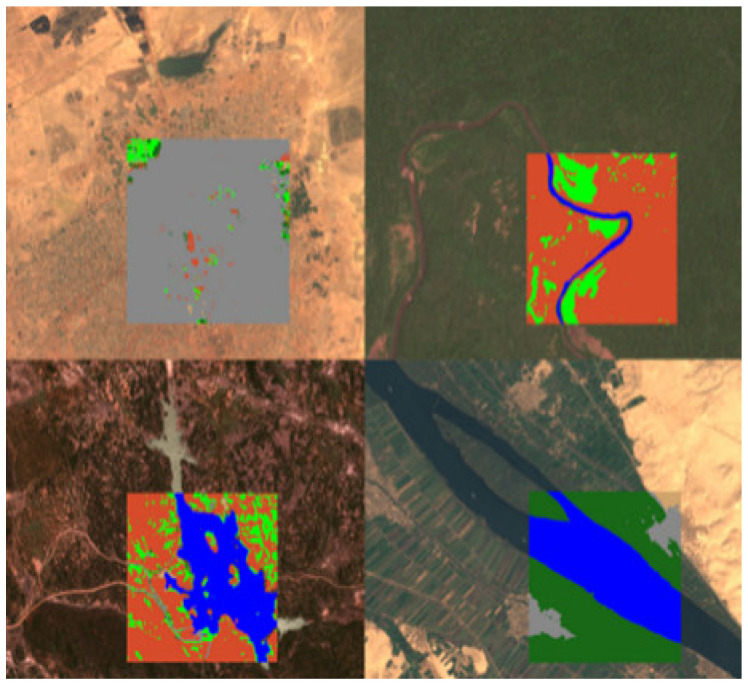
Schematic diagram of LandCoverNet.

- The Indian Pines [33,34] was created by NASA in 2015 and was the first public dataset to be proposed for land cover classification. The AVIRIS sensor (224 spectral bands) imaged Indian Pines, Indiana, and the sample dataset covered 145 by 145 pixels with 16 land cover classes and a spatial resolution of 20 m. The Indian Pines dataset is small in size but still representative of developing new algorithms and methods. For evaluating algorithms, a ratio of 80:20 or 70:30 is usually chosen to divide the training and test sets (Figure 7).
- Pavia University [34] was published in 2015, and the image was captured by a ROSIS sensor with a size of 610 × 340 pixels. There are nine different land cover classes, including pasture, bare soil, grassland, etc., containing a total of 103 spectral bands. A ratio of 80:20 or 70:30 is usually chosen to divide the training and test sets to evaluate the algorithms (Figure 8).
- LoveDA [35] is an adaptive ground cover dataset created by the RSIDEA team at Wuhan University in 2021 for land cover mapping. The 5987 images of this dataset are taken from the data collected by GoogleEarth in three areas, Wuhan, Nanjing, and Changzhou, and each image is 1024 × 1024 pixels in size with a spatial resolution of 0.3 m after correction processing. Due to the late publication of the LoveDA dataset, it has had less impact compared to some of the classic semantic segmentation datasets, such as Indian Pines and Pavia University (Figure 9).
- LandCoverNet [36] was created in 2020 as a public dataset for land cover classification for the world. These data are taken from Sentinel-1/2 and Landsat-8 multispectral satellite images acquired in 2018. This dataset has a total of 1980 samples with a size of 256 × 256 pixels and a spatial resolution of 10 m. It includes seven feature classes, namely, artificial bare ground, natural bare ground, cultivated vegetation, woody vegetation, semi-natural vegetation, water, and permanent snow/ice (Figure 10).

In recent years, LULC mapping has achieved remarkable results in various fields, such as ecological change detection and land use trend prediction, which has increased the interest of scholars in various countries in making sample datasets, resulting in an increasing number of pixel-level benchmark datasets.

Several commonly used pixel-level benchmark datasets are shown in Table 1. It can be seen that most of the datasets are hyperspectral and multispectral data, which brings diverse spectral characteristics of features to the model and thus improves the classification accuracy. However, most of the sample sets have a relatively small number of feature classes, which may be due to the high degree of similarity between some of the feature classes and, hence, their grouping together. For example, there are color similarities between artificial and natural bare ground in the LandCoverNet dataset, and without segmentation, the model’s LULC classification in new areas may be confusing, leading to a decrease In the model’s generalization ability. Also, from the table, we see that the pixel-level LULC classification datasets are small in size, which may lead to an imbalance in the number of samples contained between different categories, making the model more biased towards learning categories with a large number of samples. Thus, categories with fewer samples may be far less accurate in classification than those with more samples. Too few samples also will lead to low generalization ability of the model, as the model is trained with only a small number of samples and captures a small number of features, so it is difficult to accurately differentiate between categories on new remote sensing images.

### 3.2. Patch-Level Datasets

Instead of labeling and categorizing each pixel, patch-level datasets are produced in image segments, i.e., entire blocks of images are labeled into a scene category. These image segments have a fixed size and location and thus are similar to remote sensing scene recognition datasets. Compared to the process of making pixel-level datasets, the production of patch-level datasets is much simpler, and it does not require a lot of labor to label the images. Therefore, the disadvantage becomes very obvious: such labeling is coarse and does not capture the details inside the image blocks. Four patch-level benchmark datasets for LULC classification are described below.

**Figure 11:**
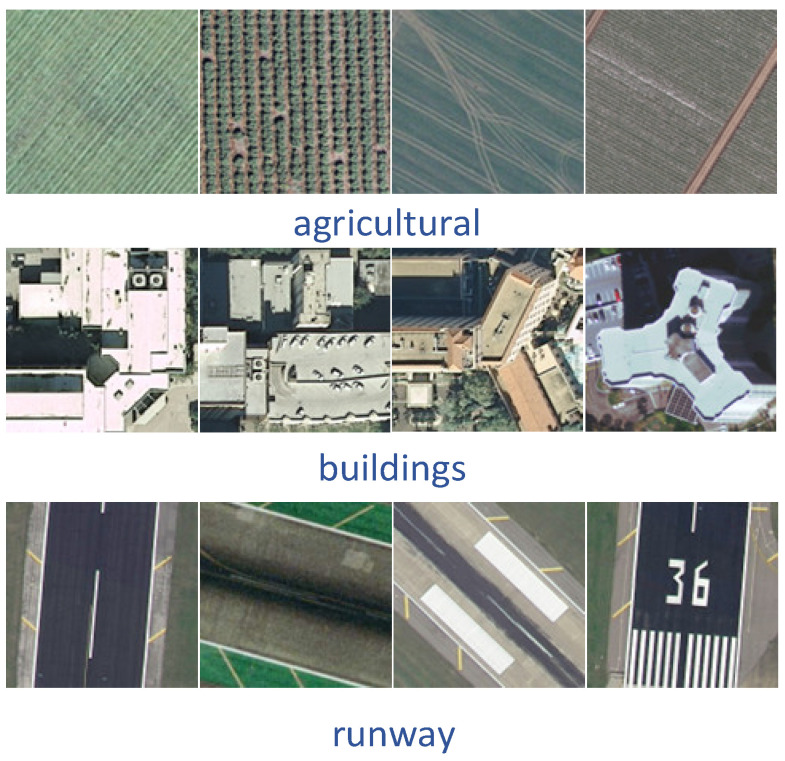
Schematic diagram of the UC Merced.

**Figure 12:**
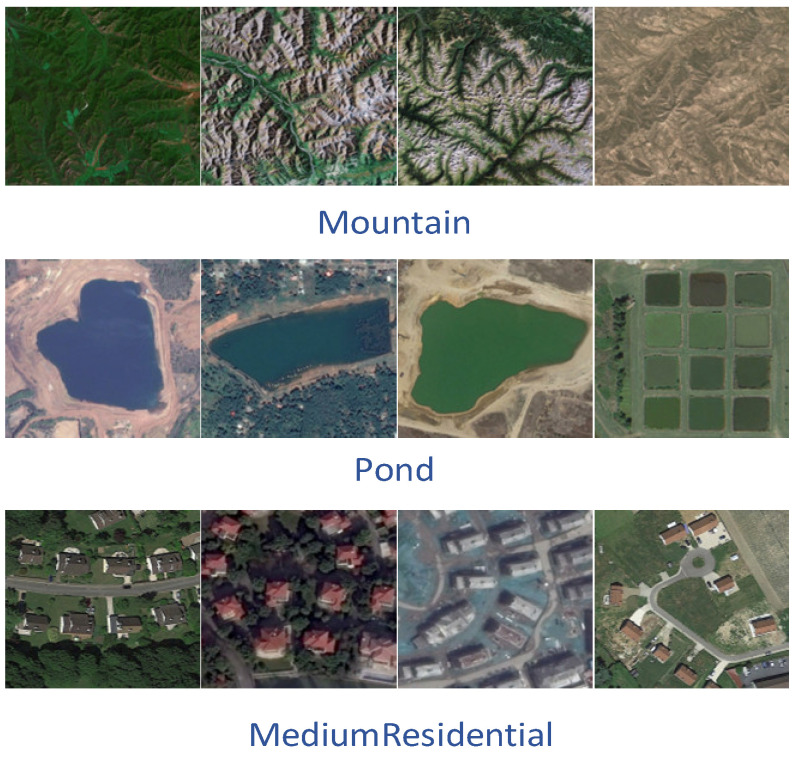
Schematic diagram of the AID.

**Figure 13:**
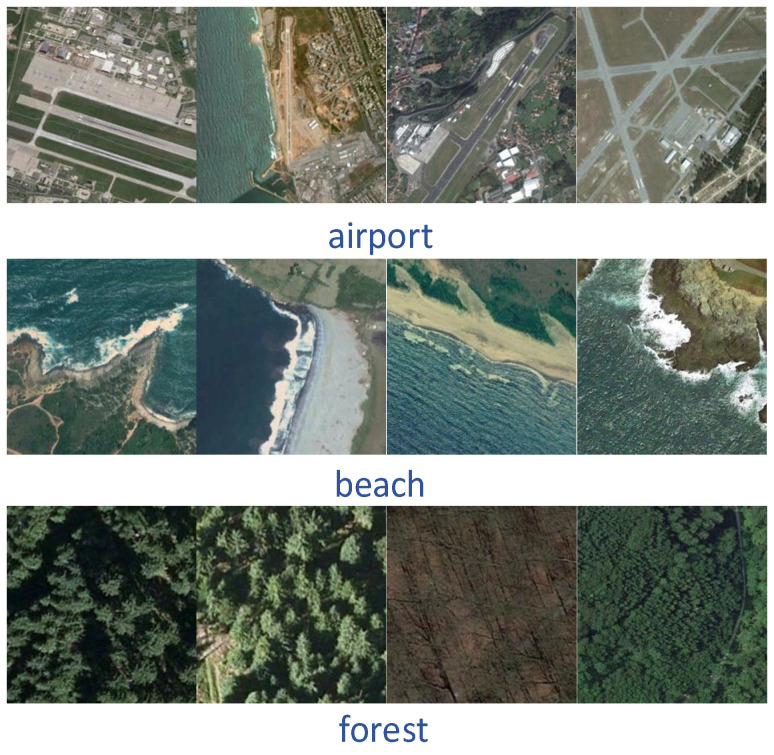
Schematic diagram of the NWPU-RESISC45.

**Figure 14:**
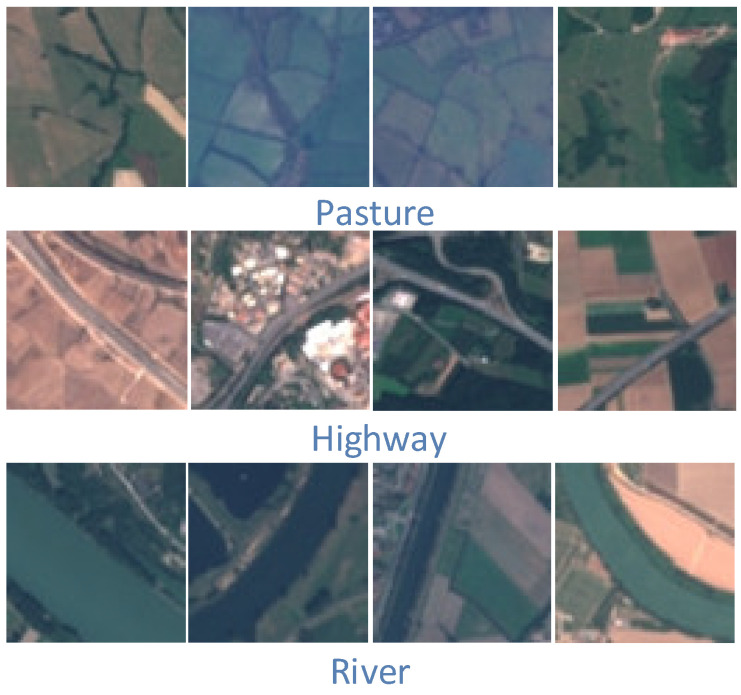
Schematic diagram of the EuroSAT.

- The UC Merced [39] dataset was proposed by researchers at the University of California, Merced, in 2010 and contains 21 scene categories. Each scene category has 100 images with a region of 256 × 256 pixels, where each pixel has a spatial resolution of 0.3 m. The 2100 images were collected from a variety of regions, including Los Angeles, Houston, and Miami, and cover a wide range of land-use types in the U.S. region. The vast majority of land use remote sensing classification experiments use the UC Merced dataset for data comparison, and again, it was one of the first datasets to be proposed. Most of the experiments divide the UC Merced dataset into an 80:20 or 50:50 ratio between training and test sets for algorithm evaluation (Figure 11).
- The AID [40] dataset was proposed in 2017, and was created by a research team from Wuhan University from images collected from Google Earth Images, covering many countries and regions around the world. The dataset contains 30 scene categories, each category contains 220 to 420 images, and the entire dataset has a total of 10,000 images, all of which are 600 × 600 pixels in size and have a spatial resolution of 8 m to 0.5 m. Because the images of the AID dataset are collected from multiple sensors, the algorithm is usually evaluated by dividing the training set and test set into 20:80 and 50:50 ratios (Figure 12).
- The NWPU-RESISC45 [41] is a dataset proposed by a research team from Northwestern Polytechnical University in 2017, with 45 scene categories, each with 700 images, all of which have a size of 256 × 256 pixels. Most of the images in this dataset have a spatial resolution of 30 m to 0.2 m, with lower spatial resolution for snow-covered mountain, lake, and island images. Because the NWPU-RESISC45 dataset contains a large number of scene categories, there exist some categories with high similarity between them, which makes it more challenging to develop new algorithms and methods (Figure 13).
- The EuroSAT [42] dataset is a large-scale dataset containing 27,000 images and was presented in 2019. It consists of images captured by the Sentinel-2 satellite and contains 13 spectral bands. A total of 10 scene categories are included, namely, cities, forests, farmland, grasslands, lakes, rivers, coasts, deserts, mountains, and industrial areas. Each category has 2000–3000 images, each with a region of 64 × 64 pixels. When evaluating the algorithm, a ratio of 80:20 is usually chosen to divide the training and test sets (Figure 14).

Table 2 lists several common patch-level datasets. As can be seen from the table, most of these datasets are dominated by Sentinel-2 (e.g., BigEarthNet, EuroSAT) and Google Earth satellite imagery (AID, RSD46-WHU, etc.), with high spatial resolution, mostly 0.3–2 m. Of these, all contain only three spectral bands, except for two datasets released in 2019, BigEarthNet and EuroSAT, which have 13 spectral bands. These extra spectral bands can provide diverse data, enabling remote sensing images to provide detailed and comprehensive information on surface features. Therefore, a number of spectral bands greater than 3 may yield more accurate classification.

These datasets mentioned above are large in scale, covering many regions and scenes around the world, with rich data diversity. At the same time, the images in these datasets have high spatial resolution and can be capable of high-precision feature classification tasks. Despite the large size of these datasets, there is still the problem of the relatively limited amount of data compared to the complexity and diversity of reality. On the other hand, some datasets, e.g., EuroSAT, have too few feature categories, making it difficult for the network to capture subtle differences between categories and reducing the generalization ability of the model.

In general, the pixel-level samples lack a large and high-quality dataset like the patch-level. Moreover, the areas covered are relatively homogeneous and only suitable for specific regions, and the generalizability needs to be improved. The patch-level dataset has fewer spectral bands, which limits the model’s ability to extract spectral information from features, thus limiting the scope of the application. Therefore, some hyperspectral and multispectral data need to be included. Hyperspectral/multispectral images can provide a more accurate estimation of mixing ratios, which leads to more accurate mixing analysis results. On the other hand, hyperspectral/multispectral imagery also allows for better differentiation of spectral characteristics between different features. The reflectance of different features at different wavelengths has unique characteristics, and hyperspectral imagery can capture these subtle differences, thus providing a better ability to identify and classify substances. This is important for applications such as LULC classification, vegetation type identification, and water monitoring.

### 3.3. Performance Indicators

The main performance metrics commonly used to evaluate LULC classification models are overall accuracy (OA), average accuracy (AA), F1-score (F1), and mean intersection and parallel ratio (MIOU) [46].

- Overall accuracy (OA) is the most used performance metric in LULC classification to measure the rate at which the model correctly predicts the samples in the dataset, which is represented by Equation (2).

(2) $$\mathrm{OA}=\frac{\mathrm{TP}+\mathrm{TN}}{\mathrm{TP}+\mathrm{TN}+\mathrm{FP}+\mathrm{FN}}$$

where TP denotes true positive, TN denotes true negative, FP denotes false positive, and FN denotes false negative.

- Average accuracy (AA) indicates the average of the rate of correct predictions by the model in each category sample, as represented by Equation (3).

(3) $$\mathrm{AA}=\frac{\mathrm{sum}(\;\mathrm{recall}\;)}{i}$$

where recall denotes the recall rate.

- F1-score (F1) is the average value after calculating the precision rate and recall rate reconciliation; the larger the F1, the better the model performance, represented by Equation (4).

(4) $$F1=2\times \frac{\;\mathrm{precision}\;\times \;\mathrm{recall}\;}{\;\mathrm{precision}+\mathrm{recall}\;}$$

where precision denotes the rate of accuracy.

- Mean intersection and unity ratio (MIOU) represents the average of the prediction accuracies of all the categories in the dataset and is commonly used to evaluate semantic segmentation, As expressed by Equation (5).

(5) $$\mathrm{MIoU}=\frac{1}{k+1}\sum_{i=0}^{k}\frac{\mathrm{TP}}{\mathrm{FN}+\mathrm{FP}+\mathrm{TP}}$$

where k denotes the number of categories and k + 1 denotes the number of categories plus the background class.

## 4. Deep-Learning-Based LULC Classification

### 4.1. Convolutional Neural Networks

**Figure 15:**
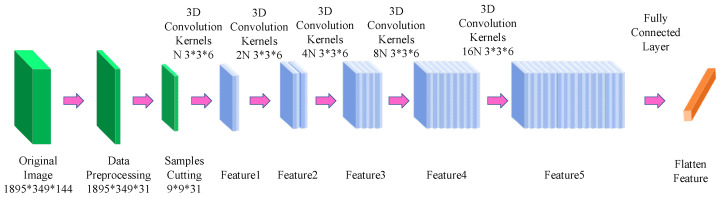
3D-CNN architecture diagram.

CNNs can effectively capture textural, spatial, edge, and contextual features of feature classes in remote sensing images and can be adapted to remote sensing images at different scales, which makes them very suitable for processing LULC classification tasks. However, the training of CNN relies on a large number of labeled samples, which is prone to overfitting and difficulty in dealing with some small-sized features in the case of scarce labeled samples. Temenos et al. [47] proposed a novel neural network framework that utilizes the SHAP deep interpreter to feed the results classified by the CNN to improve the accuracy of the final classification results. Pei [48] and others worked on the accurate classification of forest types and proposed a novel convolutional neural network, MSG-GCN. The network architecture is characterized by several features: First, to reduce the computational complexity, image features are extracted using multi-scale convolutional kernels incorporating different sensory fields. Second, to fully express the multi-scale, as well as the edge features, the authors use the Multi-scale Graph Convolutional Network (MSGCN) as a transition module. Again, to avoid the use of redundant information, the skip connection in U-Net++ is replaced with local attention. Finally, fusion is performed on the decoding layer, which synthesizes the high-level and low-level feature information and improves the accuracy of the model classification. The authors conducted comparative experiments with MSG-GCN with some state-of-the-art semantic segmentation methods, such as U-Net [49], FCN [50], and U-Net++ [51]. The results show that MSG-GCN can achieve 85.23% of the highest OA and 78.08% of the highest Kappa. Also, it can achieve 43.74% of the IoU in natural mixed forests, which is 9.34% and 10.07% higher than that of U-Net and U-Net++, and it can draw accurate maps to facilitate the management of forests. Ma [52] and others focused on developing spatial information for airborne hyperspectral data, for which a model called 3D-1D-CNN was proposed. The model can well cope with the challenges of feature extraction in complex urban areas, especially those affected by cloud shadows. Specifically, all the parameters extracted from the hyperspectral data are fused and partitioned into many stereo blocks to be passed to the 3D-CNN classifier, which contains texture features, spectral composition parameters, and vegetation indices, and their fusion provides rich information for feature extraction, enabling the model to extract features of complex urban areas more accurately. In the comparison experiments of methods such as 3D-1D-CNN, 1D-CNN, and 3D-CNN (see Figure 15), it was found that 3D-1D-CNN achieved the highest OA of 96.32%.

**Figure 16:**
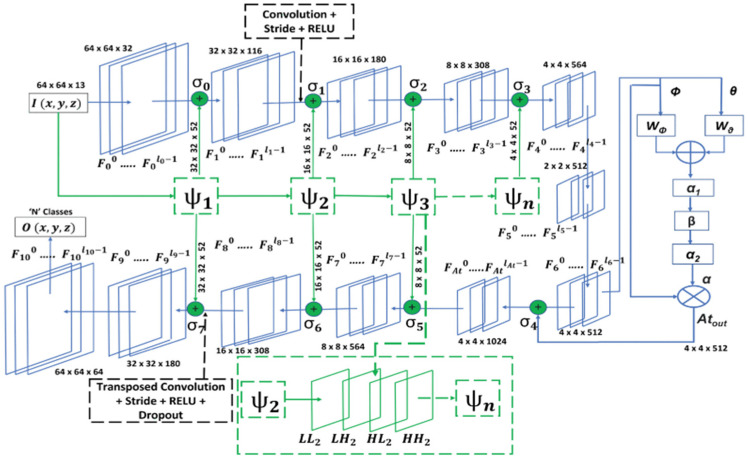
WIANet network architecture.

For complex scenarios, Khan et al. [53] proposed a multi-branch framework consisting of two deep learning networks, DenseNet [22] and FCN. The DenseNet network is used to extract the contextual features of the input image, and FCN is used to extract the local features of the input image. Finally, the features extracted by the two deep learning networks are fused and optimized with loss function. The network framework achieves the highest OA of 99.52%, 96.37%, and 94.75% on the UC Merced [39], Siri-WHU [54], and EuroSAT [42] datasets. Compared to methods such as GoogleNet, ResNet-50, MOEA, and ResNet-101, the authors’ method achieves higher accuracy and superior performance. Xia et al. [55] proposed a deep learning model based on unsupervised domain adaptation (UDA), which is capable of adapting to large-scale land cover mapping. Specifically, it solves the deep convolutional neural network (DCNN) biased mislabeling problem by using Siamese networks [56], and combines dynamic pseudo-label assignment and class balancing strategies, thus realizing adaptive domain joint learning. The authors compared their method with several UDA methods applicable to large-scale cities, such as AdaptSeg [57], AdvEnt [58], CLAN [59], and FADA [60], and after incorporating dynamic pseudo-label assignments, respectively, their method achieves the highest OA under sparse samples with 81.14%, 51.20%, 40.81%, and 40.81% of the OA, mF1, and MIOU. The highest OA, mF1, and MIOU of 80.85%, 55.33, and 43.99 were achieved with dense samples. Wang [61] and his team focused on research on crop classification, and they found that the existing crop classification methods could not be applied to multi-scenario challenges, so they proposed a deep learning method called Cropformer, which is based on a new type of transformer and combines it with convolutional neural networks. The method differs from existing crop classification methods in that it accomplishes both global and local feature extraction. The research team utilized Cropformer for grouping crop experiments in multiple scenarios. Experimental results show that Cropformer is more accurate and efficient than existing crop classification methods. It can extract additional information from unlabeled data to build up a priori knowledge for later crop classification. This a priori knowledge makes Cropformer better at handling the task of feature classification and more widely applicable. Singh et al. [62] developed an attention-based convolutional neural network (WIANet) architecture (see Figure 16) that overcomes the lack of spectral context modeling in CNNs when processing multispectral images. The authors introduced the Wavelet Transform (WT) [63], as well as an attention mechanism to the U-Net-based architecture to improve the network’s ability to discriminate spectral features, with the aim of more accurately distinguishing between classes whose spectral features have a high degree of similarity. Their methods achieved 92.43%, 91.80%, and 84.69% OA on the EuroSAT [42], NaSC-TG2 [64], and PatternNet [65] datasets, respectively, and achieved F1 on the EuroSAT dataset, which is higher than methods such as EfficientNet-B1 [66], RegNetX120 [67], RegNetY120 [67], and others.

### 4.2. Generating Adversarial Networks

**Figure 17:**
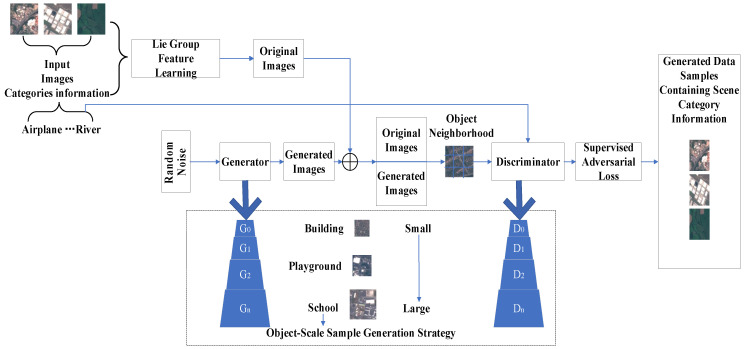
Novel Lie Group generative adversarial learning network.

GAN is widely used for land cover classification in the case of sparse samples due to its unique adversarial mechanism that generates realistic images to act as samples and to increase the training samples of the network, thus improving the accuracy of classification. GAN can generate images for categories with fewer sample data, solving the problems of low classification accuracy and overfitting in some categories caused by unbalanced sample data. In addition, the images generated by GAN are cleaner, reducing the negative impact of noise in the original data. Ansith et al. [68] changed the input to the generator in the GAN by using an encoder to convert all remote sensing images in the dataset into potential vectors, replacing the original potential vectors generated from the network noise signal. This GAN structure, combined with an encoder, recognizes more details and thus achieves higher accuracy even when the samples are sparse. The authors’ method achieved 97.68% of the highest OA and 97.43% of the F1 on the AID dataset, and 95.68% of the OA and 95.38% of the F1 on the UC Merced dataset. Ma [69] and others proposed a generative adversarial network called SPG-GAN, which aims to allow GAN to generate samples with category labels and thus achieve higher classification accuracy. They trained the network in the direction of labeling information by including label information on the inputs of the generator and the discriminator. Moreover, the generator sample module is added to the generator and discriminator iterations, respectively, so that the final labeled samples generated are of higher quality and rich in more features and spatial details. SPG-GAN achieves the highest OA of 79.88% and 65.13% on the UC Merced and AID datasets, respectively. Miao et al. [70] introduced an additional convolution (involution) layer on the generator and discriminator layers of the GAN, respectively, to strengthen the feature extraction capability of the network. To narrow the gap between labeled and unlabeled samples, the authors introduced a consistency loss function on the Siamese network that performs semi-supervised classification. The method achieved the highest OA of 94.25% and 92.03% on the AID and RESICS-45 datasets, respectively. Xu et al. [71] proposed a new type of generative adversarial network called Lie Group (as shown in Figure 17) to cope with problems such as the decline in the learning ability of deep learning models due to the scarcity of samples. Like the method proposed by Miao et al. [70], the inputs of this network model are also composed of category information and data samples such that the newly generated data samples contain category information. The difference is that they introduced an object-scale sample generation strategy to ensure that the new data samples contain more feature information. Experiments show that the network still achieves highly accurate classification even with sparse samples. Because the scale of the receptive field of the FCN network is fixed, it is difficult for the model to judge objects that are much smaller than the current receptive field scale, thus causing misclassification. For this reason, Wang et al. [72] proposed a GAN that combines a multi-scale receptive field, which improves the performance of the model in the case of fewer labeled samples by introducing a space pyramid pooling (ASPP) module to obtain the multi-scale features and by utilizing semi-supervised training of the GAN, which alleviates a large amount of labeling and improves the performance of the model in the case of fewer labeled samples. This improved GAN achieves 75.25%, 78.66%, and 80.71% MIOU on the CCF2015 dataset with 1/8, 1/4, and 1/2 the amount of data, respectively, higher than several state-of-the-art semantic segmentation methods compared with it.

### 4.3. Recurrent Neural Networks

Although convolutional neural networks are most common in LULC, RNNs are better in special cases, especially when dealing with time-series data, where they can accurately capture the sequence information. The RNN is hidden in each time step due to its special hidden state, and this hidden state is propagative and can be passed from the previous time step to the next, whereby the RNN can capture rich contextual information. In addition, RNN can adaptively extract features when training data, eliminating the complicated process of designing a feature extractor. Ma et al. [25] incorporated Long Short-Term Memory (LSTM) into the RNN network to improve the efficiency of RNN computing similarity. Specifically, the method of object segmentation makes the network search for the target pixel from the whole map to the segmented map, then filters out the line segments with similar features, and finally extracts similar pixels from these line segments, which greatly shortens the time of similarity calculation. Also, for target pixels, the study considers both local and non-local segments, bringing more spatial information to the network. Tang et al. [73] proposed a recurrent neural network called DRRNN, which learns iteratively by utilizing class correlation features (CCFs) [74], and at each iteration, CCFs use spatial information to correct misclassification, in this way learning repeatedly until they reach an optimal state to discontinue the iteration. Tao [75] and others combined CNN and RNN to propose a network called SIC-Net, which allows the network to utilize the spatial information of remote sensing images to mine more discriminative features. SIC-Net is divided into two parts, the first part is used to convert inter-patch context information into inter-pixel context information, and the second part is used to model inter-pixel context information and focuses on the extraction of local and remote spatial features. This method has a great advantage in scene classification in the case of spectral confusion. Sohail et al. [76] also considered combining RNN with CNN and proposed a new neural network called MulNet. It is composed of three parts: the first part learns multi-scale local spectral spatial features of hyperspectral images by introducing a three-dimensional residual network (3DResNet), the second part employs a Feature Fusion Module (FFM) to aggregate these spectral spatial features that have been sampled at different ratios, and the third part of the RNN generates discriminative features based on these fused features as a way to achieve more accurate classification. MulNet achieved the highest OA of 91.45% and 87.15% on the Pavia University and Salinas datasets, respectively. Zhang et al. [77] integrated the non-local spatial sequence (NLSS) method into RNN and proposed a network called NLSS-RNN. The network is divided into three parts, the first part extracts low-level features from hyperspectral images. The second part extracts Local Spatial Sequence (LSS) features from low-level features using the NLSS method and preserves NLSS information. In the third part, the LSS features containing NLSS information are used as inputs to the RNN network as a way to generate high-level features. Finally, the advanced features are fed into the classifier for classification. This network that generates advanced features from low to high achieves the highest OA of 98.75%, 99.77%, and 97.23% on the Indian Pines, University of Pavia, and Salinas datasets, respectively, which is better than some of the state-of-the-art classification networks.

### 4.4. Autoencoder

Autoencoders have excellent downscaling and are, therefore, often used to process hyperspectral data in LULC. AE has a unique encoding–decoding mechanism to mine the potential features in the original data, as well as to reconstruct the original data, which helps the model to learn the data at a deeper level to generate discriminative features with higher discriminative properties, thus realizing more accurate classification. As an unsupervised classification method, AE is also often used in the case of limited labeled samples, which can learn feature characteristics of features on unlabeled samples and then use the unsupervised pre-training results for supervised classification, effectively alleviating the problem of sample scarcity. Ibañez et al. [78] proposed a new spectral converter (MAEST) to alleviate the state of transformer networks where it is difficult to properly characterize continuous spectral features due to the factor of noise. MAEST is composed of a reconstruction path and a classification path. The role of the reconstruction path is to reconstruct the original image using the AE. The encoder in the AE is used to extract the potential features of the unmasked spectra, and the decoder uses the potential features to reconstruct the masked spectral information. At the same time, the encoder can extract the most robust spectral features after this process to eliminate the negative effects of noise. The role of the classification path is to integrate the features obtained from the reconstruction path onto the transformer network and utilize the more robust features for more accurate classification. MAEST achieved the highest OA of 84.15%, 91.06%, and 88.55% on the Indian, Pavia University, and Houston 2013 datasets, respectively. Chen et al. [79] proposed a novel stacked autoencoder for processing Polarized Synthetic Aperture Radar (PolSAR) images, called MDPL-PolSAR. Specifically, a multilayer projective dictionary pair learning (MDPL) was developed to extract high-dimensional features from PolSAR, and then these high-dimensional features were used as inputs to the SAEs, which were adapted and learned by the SAEs to acquire the features with high discriminative properties, thus realizing more accurate classification. Also, for PolSAR images, Liu et al. [80] developed a novel autoencoder SAE_MOEA/D, which is a combination of the Multi-Objective Evolutionary (MOEA) algorithm developed by the authors and a variant of SAE. MOEA can adaptively mine the optimal parameters, eliminating the time-consuming step of human exploration of the optimal parameters. Variants of SAE can adaptively construct the number of network layers according to the dataset to accommodate different PolSAR images. The method is robust and largely eliminates time-consuming and laborious work. Mughees et al. [81] proposed a novel classification method based on the spectral space of hyperspectral images (HSI). The method utilizes SAE as a classifier to extract the spectral features of HSI to obtain deeper spectral features. The space is then segmented using the Hidden Markov Random Field (HMRF) technique to obtain spatial features. Finally, the spectral and spatial features are fused to achieve more accurate classification. The method achieved 98.72% and 90.08% OA on the Pavia University and Indian Pine datasets, respectively. Mughees et al. [82] also improved the SAE and proposed a novel network called HVSAE. This network can adaptively segment the spatial structure of HSI, and similar spectral information is preserved in the segmented structure. These segmented spatial structures are then processed using SAE to obtain more discriminative features based on the similar spectral features in them. HVSAE achieved 90.08% and 98.98% OA on Indian Pine and Pavia University datasets, respectively. Chen et al. [83] developed a novel dimensionality reduction algorithm called SPCA, which allows the network to effectively extract spatial features in HSI. The authors used two spatial neighborhoods, one large and one small, to extract the spatial information; the large spatial neighborhood ensured sufficient spatial information, while in the small spatial neighborhood, spatial information was effectively utilized to reduce misclassification. The spectral-spatial features extracted by SPCA were then classified by SAE. The method achieved 99.27% OA on the Pavia University dataset. Mughees et al. [84] proposed a novel HSI classification method. The method consists of three parts. The first part utilizes SAE to extract deep spectral features of HSI. The second part utilizes the boundary leveling technique to segment the HSI and extracts the spatial features of the HSI in the process. The third part uses the majority voting (MV) strategy to fuse the spectral and spatial features before classifying them. The method achieves 89.35% and 98.64% OA on Pavia University and Indian Pine datasets, respectively.

## 5. LULC Challenges

1. Data diversity: Remote sensing data may change according to different times, different regions, different sensors, different climates, etc., so our model needs to be constantly updated to adapt to the variability caused by data diversity.
2. Category imbalance: When making LULC datasets, some categories will have too many or too few data samples due to factors such as differences in geographical distribution, human intervention, and labeling difficulties. Therefore, the model tends to favor the categories with more data when training these data, while the categories with fewer samples lead to poorer classification accuracy due to too few training samples.
3. Sample labeling problem: The labeling of LULC training samples not only requires researchers to have a high level of expertise and be familiar with the characteristics of the vegetation, urban, and water categories, it also requires a lot of manpower and time to classify the category areas and classifications. Some features are highly similar to each other, e.g., swamps and wetlands, and researchers may make subjective errors in judgment that lead to relatively fuzzy boundary delineation between categories, thus reducing the classification accuracy of the model.
4. Generalization ability of the model: A model may achieve high classification accuracy in a specific region; however, if it is changed to a region or area in other countries, the generalization ability of the model will be reduced due to different distribution of features, differences in the labeling of the samples, and differences in the characteristics of the categories, among other factors.
5. Contextual modeling: Remote sensing data are spatially correlated, i.e., there are dependencies between data in nearby areas. However, traditional deep learning methods do not mine the contextual information between data well, making the model unable to learn and understand the data comprehensively, which reduces the classification accuracy of the model.

## 6. LULC Classification with Limited Samples

The problem of limited training samples is one of the main challenges faced in LULC classification, and in this section, we will discuss the solutions to this challenge in terms of four methods: transfer learning, data augmentation, weakly supervised learning, and active learning.

### 6.1. Transfer Learning

**Figure 18:**
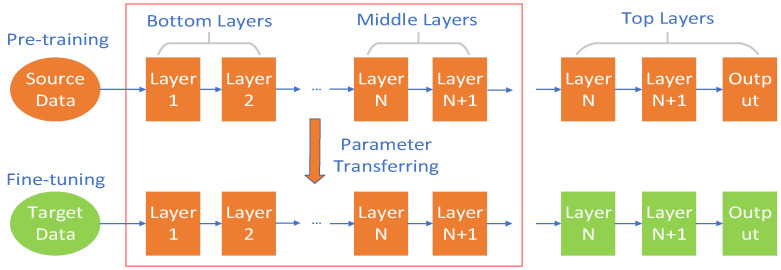
Transfer learning illustration.

Transfer learning (TL) is the application of knowledge learned in the old domain (source domain) to the new domain (target domain), which compensates for the negative impact of sample scarcity and improves the generalization ability of the model, leading to more accurate classification. Therefore, TL is often used to deal with LULC under limited samples. Domain adaptation (DA), as a subfield in TL, is even more favored by researchers because it can solve the discrepancy problem between the source and target domains. Liu et al. [85] proposed a DA-based SFnet-DA network to solve the problem of insufficient labeled samples in practical applications. Specifically, an adversarial training consisting of domain classifiers and feature extractors was designed so that the parameters obtained from training the labeled dataset can be directly used for the prediction of unlabeled images. SFnet-DA achieves 97.13% F1 on the WHDLD dataset, which is higher and faster to process than excellent semantic segmentation networks such as U-Net and Deeplab V3+. Traditional DA methods make it difficult to balance the critical classes in the target domain, which may result in some classes in the target domain lacking labeled samples for training, so an unsupervised DA method was proposed by Soto et al. [86]. Specifically, a pseudo-label generation scheme called CVA was designed, with which the feature alignment of the target and source domains is balanced to avoid over-biasing to one category. Bai et al. [87] proposed a novel model that enables DA to directly align features in the source and target domains, which contains two DA branches and a semantic segmentation network. The two DA branches, one for adapting the representation space and one for adapting the prediction results, create feedback on each other to obtain more accurate classification results. Figure 18 illustrates the transfer learning.

### 6.2. Data Augmentation

**Figure 19:**
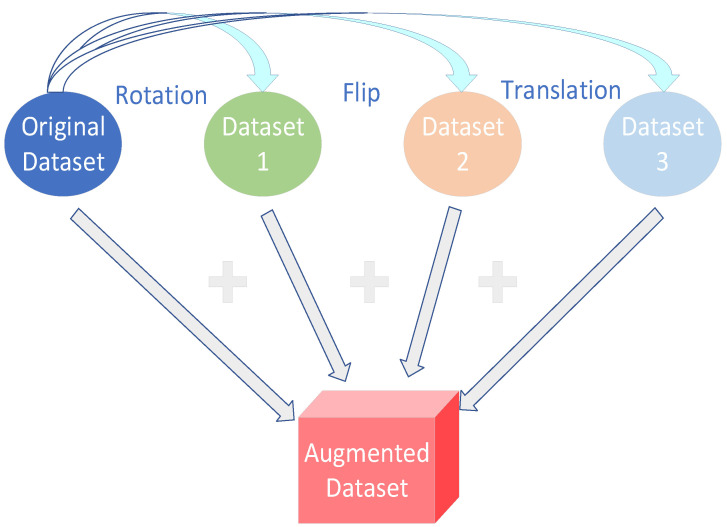
Data augmentation illustration.

Data augmentation is one of the important means of solving the finite sample problem, which involves various transformations such as rotating, scaling, and cropping of the original data as a way of generating new training samples and increasing the training volume of the model. In addition, data augmentation makes the training samples more diverse and improves the generalization ability of the model. Scott et al. [88] trained a DCNN model by augmenting and expanding the dataset with various transpositions and rotations of the training samples to obtain an upright view of each category. Experiments showed a significant improvement in classification accuracy using the data-augmented dataset. Yi et al. [89] proposed a novel data augmentation method that optimizes the network model by adding an attention mechanism to the GAN, replacing the loss function and adding a convolutional kernel, which enhances the GAN’s ability to learn global features, improves the stability of the model, and results in a higher quality of samples generated by the GAN. Figure 19 illustrates the data augmentation.

### 6.3. Active Learning

**Figure 20:**
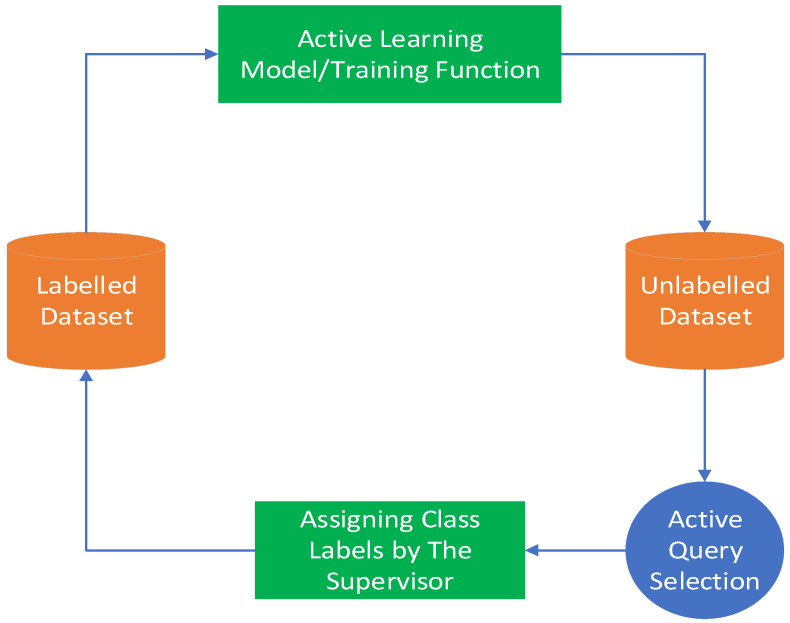
Active learning illustration.

As a kind of iterative learning, active learning (AL) adaptively selects higher quality samples from unlabeled samples to train the model and improve the performance of the model and iterates by selecting informative and low redundancy samples from them for labeling, thus reducing the need for training samples. Haut et al. [90] proposed a new model that is composed of Bayesian convolutional neural network (B-CNN) and AL. B-CNN can be adapted to train with fewer samples and extracts useful data from CNNs with different architectures, preventing the generation of overfitting and improving the robustness of the network. One of the roles of AL is to use the probability function to analyze those unlabeled samples and extract a lot of useful information for the model. Lei et al. [91] proposed a deep network method incorporating AL to overcome the problem of the limited number of training samples due to the complex and tedious labeling work of HSI. The network extracts uncertainty information from unlabeled samples, which is then incorporated into the original training samples for augmentation purposes and constitutes a new dataset. Experiments show that the method performs well on several public datasets. Figure 20 illustrates active learning.

### 6.4. Weak Supervision Methods

Traditional deep learning methods need to rely on a large number of training samples to achieve accurate classification. However, the labeling of remote sensing images is time-consuming and labor-intensive, and the limited training samples make it difficult for the model to capture deep and complex relationships. Therefore, weakly supervised learning, which is less dependent on labeled samples, becomes one of the most effective methods to solve the sample scarcity problem. In the following sections, we will explore the use of zero-shot learning and few-shot learning methods to deal with the limited sample problem in LULC.

#### 6.4.1. Zero-Shot Learning

Zero-shot learning (ZSL) allows knowledge migration, i.e., learning semantic information from visible categories and transferring it to invisible categories, thus reducing the need for labeled samples. Li et al. [92] proposed a novel zero-shot method called ZSSC, which constructs a guided graph through semantic vectors to sort out the connections between visible and invisible categories. A label propagation algorithm incorporating unsupervised domain adaptation was developed for migrating the knowledge learned from visible categories. The results show that this method is significantly better than other ZSL-based methods. Li et al. [93] proposed an end-to-end ZSL approach that bridges the class structure gap between the semantic space and the visual image space, allowing the model to efficiently utilize semantic representations to classify visual samples. An interpretable constraint was designed to improve the robustness of the network and make the network learning more stable. After a large number of experiments, it was shown that the method can effectively solve the problem caused by the scarcity of labeled samples.

#### 6.4.2. Few-Shot Learning

**Figure 21:**
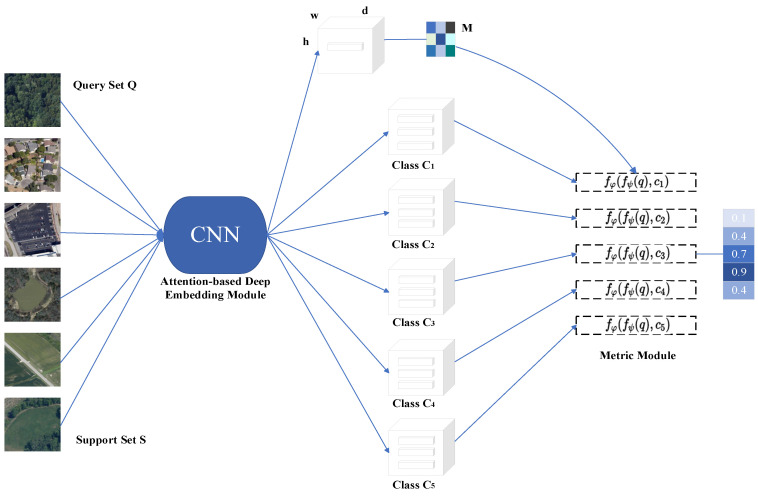
DN4AM architecture.

Few-shot learning aims to utilize a very small number of training samples to achieve effective training and fast learning. Chen [94] and others worked on how to improve the accuracy of scene classification in the presence of sample scarcity. To this end, they proposed a few-shot classification method called DN4AM. Specifically, the influence of semantically irrelevant objects is weakened by using the attention graph associated with each category, which makes the model more focused on classification-related information and improves the accuracy of model classification. The authors used the NWPU-RESISC45, UC Merced, and WHU-RS19 datasets to evaluate the performance of DN4AM (Figure 21). The results show that DN4AM demonstrates excellent performance and achieves high-precision classification even when the labeled samples are scarce. Jiang et al. [95] proposed a graph-based few-shot learning method that is composed of two models: feature learning and feature fusion. The feature learning model is used to transform the extracted multi-scale features into graph-based features, allowing the model to better utilize the spatial relationships between remote sensing images. Feature fusion models are used to integrate graph-based multi-scale features to obtain more semantic information as a way to achieve more accurate classification in few-shot conditions.

## 7. Prospects

Combined with the most advanced deep learning technologies and innovations in recent years, LULC classification will face the following important trends:

- Cross-modal data fusion: With the rapid development of deep learning methods in the field of LULC classification, more research will be conducted in the future to fuse effective information from multiple modalities (e.g., texture, spectral, and temporal information, etc.) of remote sensing imagery and combine them with deep learning models to improve the accuracy and robustness of the network model classification.
- High-resolution data: The resolution of remote sensing image data will increase in the future, which puts more requirements on LULC classification. Therefore, one of the focuses of future research is to develop better algorithms and models to adapt to high-resolution data to capture the details and changes in feature characteristics more accurately.
- Expert knowledge: Although deep learning and other technologies have achieved excellent results in LULC classification, it is still very important to integrate the experience and knowledge of human experts. It can not only be converted into an interpretable form of the model to improve the explanation ability of the classification results but also correct the misclassification of the model to achieve higher robustness and accuracy, which is in line with the needs of practical applications.
- Transfer learning and adaptive learning: The combination of the two can help solve the problem of low generalization ability of models due to domain differences. Future research can explore how to narrow the gap between the source and target domains due to transfer learning and optimize the model using adaptive learning to enable higher adaptation on the target domain for higher classification performance and generalization ability.

In the future, LULC classification will continuously improve the generalization ability and robustness of the model, as well as the accuracy and explainability of the classification with the help of cross-modal data fusion, high-resolution data, transfer learning and adaptive learning, among other technical means. This will provide more accurate and reliable information support for resource management, environmental protection, and sustainable development.

## 8. Conclusions

LULC classification plays an important role in urban and rural land planning, disaster prediction, monitoring of environmental change, and sustainable development. Early LULC classification methods based on low-level feature extraction unfolding can achieve good results on small-scale datasets, yet have limited results for large-scale as well as complex datasets. Therefore, as the variety and number of LULC sample datasets continue to expand, deep-learning-based classification methods have stepped onto the stage, bringing automation, adaptability, and accuracy to the LULC task, allowing us to better learn and manage surface resources. In this paper, we briefly introduce some commonly used deep learning models, including; CNN, RNN, AE, GAN, and FCN. After that, we systematically summarize the common public datasets commonly used for LULC classification in terms of samples and categorize them as pixel-level datasets and patch-level datasets, respectively. We also summarize the evaluation criteria used in LULC classification. In addition, we overview the excellent performance demonstrated by deep learning methods in LULC classification and briefly clarify the advantages and disadvantages of each method. Despite the fact that DL is very effective in handling LULC classification tasks, there are some limitations and challenges faced by LULC classification that prevent DL from realizing its full potential. Thus, in this paper, we summarize the challenges faced by LULC classification, elaborate on the treatment scheme in the case of scarcity of labeled samples, and finally point out the corresponding future directions to face these challenges.

## Figures and Tables

**Table 1:** Pixel-level benchmark datasets.

Dataset	Data Sources	Number	Image Dimensions	Spatial Resolution	Number of Bands	Number of Categories	Year
SPARCS [37]	Landsat8	80	1000 × 1000	30	10	7	2014
LoveDA [35]	GoogleEarth	5987	1024 × 1024	0.3	3	7	2021
Kennedy Space Center [34]	AVIRIS	1	614 × 512	18	224	13	2014
Pavia University [34]	ROSIS	1	610 × 340	1.3	103	9	2015
GID [38]	GF-2	150	6800 × 7200	1/4	4	15	2020
Indian Pines [33,34]	AVIRIS	1	145 × 145	20	224	16	2015
LandCoverNet [36]	Sentinel-1/2 Lantsat-8	1980	256 × 256	10	10	7	2020

**Table 2:** Patch-level benchmark datasets.

Dataset	Data Sources	Number	Image Dimensions	Spatial Resolution	Number of Bands	Number of Categories	Year
BigEarthNet [42,43]	Sentinel-2	590,326	120 × 120	10	13	43	2019
RESISC45 [41]	WorldView-2	31,500	256 × 256	2	3	45	2016
EuroSAT [42]	Sentinel-2	27,000	64 × 64	10/20/60	13	10	2019
AID [40]	Google Earth	10,000	600 × 600	2	3	30	2017
UC MERCED [39]	Aerial imagery	2100	256 × 256	0.3	3	21	2010
OPTIMAL-31 [44]	Google Earth	1860	256 × 256	-	3	31	2019
RSD46-WHU [45]	GoogleEarth, Tianditu	117,000	256 × 256	0.5–2	3	46	2017

## Data Availability

The training data presented in the study are openly available at https://aistudio.baidu.com/datasetoverview.
